# Editorial: Bone metastasis in the milieu of osteoimmunology

**DOI:** 10.3389/fimmu.2023.1265434

**Published:** 2023-08-07

**Authors:** Gunjan Sharma, Marco Ponzetti, Jawed A. Siddiqui

**Affiliations:** ^1^ Department of Biochemistry and Molecular Biology, University of Nebraska Medical Center, Omaha, NE, United States; ^2^ Department of Biotechnological and Applied Clinical Sciences, University of L’Aquila, L’Aquila, Italy; ^3^ Fred and Pamela Buffett Cancer Center, University of Nebraska Medical Center, Omaha, NE, United States

**Keywords:** bone metastasis, osteoimmunology, immune checkpoint inhibitor, osteosarcoma, tumor microenvironment (TME)

Bone metastasis severely hampers the survival of advanced cancer patients, and the nature of treatments is generally restricted to primary tumors. The mechanisms causing this discrepancy between therapy effectiveness in the different sites depend on several factors, a crucial one being the microenvironment. In particular, a pivotal role of the immune system has emerged in the last few years. However, despite great advances in the field, there is still much to be done to understand and exploit the immunological side of bone metastases to fight cancer. This prompted us to initiate this Research Topic entitled “*Bone metastasis in the milieu of Osteoimmunology*” to understand more about cancer and various immune aspects in the bone microenvironment (**Figure 1**). A total of nine articles were published in this Research Topic, including five original research papers (Talbot et al., Li et al., Yang et al., Guo et al., and Chang et al.), three review articles (Yu et al., Tong et al., and Kähkönen et al.), and one case report (Asano et al.). One original research and one review article containing B7-H3-CAR T Cells, Cuproptosis-related lncRNA, cancer stem cell-related genes, and chemoresistance in turn of immune modulation in osteosarcoma (OS). One research and one review article deal with the drug repurposing and interleukin-1 family in prostate cancer bone metastases. Two more articles, one original research, and one case report, have been included in the topic; both studies are associated with immunotherapy in bone-metastatic patients with advanced non-small cell lung cancer and renal cell carcinoma.

**Figure 1:**
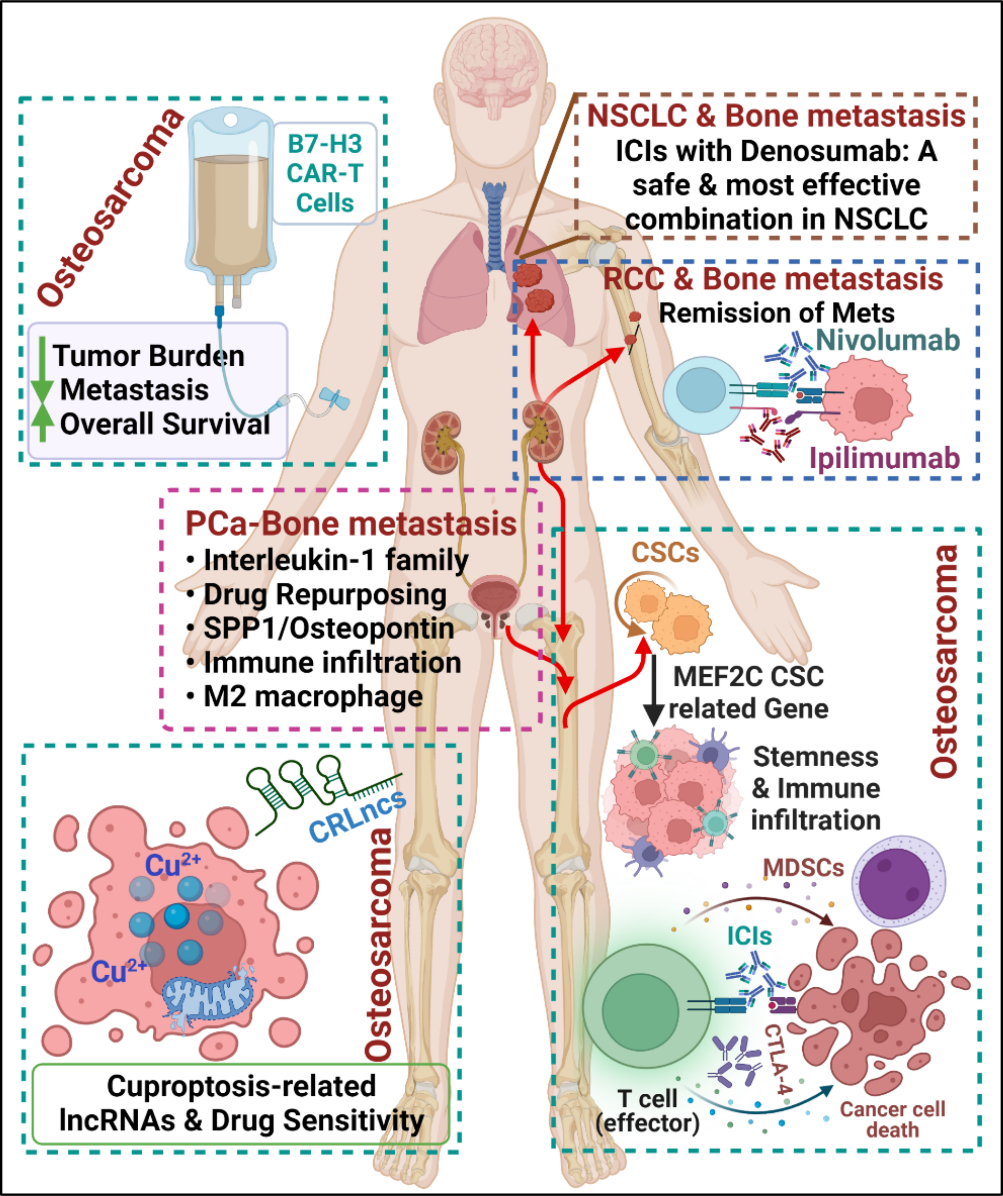
Cartoon summarizing the contributions submitted to the Research Topic, including osteosarcoma and bone metastasis in PCa, NSCLC, and RCC, showing a multidisciplinary approach, attractive osteoimmunological targets, and therapeutic outcomes in preclinical and clinical settings. (Created with biorender.com).

Innovative technologies and ideas are leveraging the advancement of our knowledge, specifically cancer and osteoimmunological aspects in the bone microenvironment. Talbot et al. study equipped with a novel orthotopic (tibial osteotomy) implantation procedure for OS cells where B7-H3, an immune regulatory protein, crucially participates in spontaneous metastasis. B7-H3 targeted Chimeric antigen receptor-T (CAR-T) cells (injected via tail vein) therapy reduces the tumor burden and metastatic spread to the lungs, thenceforth enhancing the survival of NOD scid gamma mouse (NSG) mice. Another original research by Yang et al. included in this Research Topic is based on the long noncoding (lnc) RNA link to novel copper-mediated cell death (cuproptosis) in the prognosis of OS. This article aims to correlate the status of cuproptosis-related lncRNAs (CRLncs) with the survival outcomes of OS patients. Analysis of OS transcriptome and clinical dataset from The Cancer Genome Atlas (TCGA) revealed three high-risk CRLncs and three low-risk CRLncs involved in OS prognosis and immune microenvironment. In a drug-sensitive study, authors found four potent drugs: AUY922, bortezomib, and Z.LLNle.CHO is sensitive to low-risk and lenalidomide for the high-risk OS group.

Furthermore, Guo et al., by using consensus clustering analysis, identified 25 cancer stem cell-related genes and classified OS into three (CSC cluster A, B, and C) molecular subtypes. Among 25 CSC-related genes, authors emphasized MEF2C as a significant player in immune infiltration and tumor cell stemness which correlated with patient survival. A recent study in bladder cancer also revealed MEF2C as a prognostic factor and putative role in immune modulation (1). Moreover, the authors established a unique CSC scoring system that could be beneficial in assessing tumor-microenvironment (TME) immune infiltration and for personalized immunotherapy of OS patients.

The role of chemokines and cytokines is highly correlated with tissue-specific tropism, niche formation, and colonization of cancer cells (2). However, interleukins (ILs) are well known for the inflammatory response in various diseases and tumor microenvironments. Tong et al. highlight the pivotal role of the IL-1 family in prostate cancer (PCa) progression and bone metastasis. As PCa exhibits the highest incidence of bone metastasis, authors attractively connect IL-1 family-mediated inflammation with PCa bone metastasis progression. They have covered several ILs’ functions as anti- and pro-tumorigenic activity. Additionally, the authors provide a glimpse of ILs’ diverse role in colonization, dormancy, reactivation, angiogenesis, and bone remodeling. In a transcriptome-based multiplex drug repurposing study, Chang et al. validated a new scheme to screen specific candidate compounds to prevent bone metastasis of castration resistance prostate cancer (CRPC) patients. After a battery of comprehensive analysis, two compounds, namely CID 190453/mulberroside C and CID 78177919/terrestrosin D, show selective and potent binding with Secreted phosphoprotein 1 (SPP1), also known as Osteopontin, that can regulate the M2 macrophage-associated PCa-bone metastatic genes. Elevated expression of SPP1 is highly correlated with immune cell infiltration and poor survival in various cancers (3).

Li et al. performed a study on 171 non-small cell lung cancer (NSCLC)-bone metastatic patients and categorized them into four groups. Among all groups, DI, i.e., patients receiving a combination of denosumab with immune checkpoint inhibitors (ICIs), reveals safe, highest drug efficacy and survival of bone metastatic NSCLC patients with minimal side effects. Recently, in a clinical trial, dostarlimab, an anti–PD-1 monoclonal antibody, showed complete remission in all advanced rectal carcinoma patients (4).

In a series of therapeutic efficacy of ICIs against bone metastasis, Yu et al. summarize the recent studies on various mechanisms of chemoresistance and vulnerable targets, including myeloid-derived suppressor cells (MDSCs). In this review, authors emphasize the status of different immunotherapeutic targets such as programmed death-ligands/receptors (PD-1/PD-L1) and cytotoxic T lymphocyte-associated protein 4 (CTLA-4) and clinical trials of ICIs for OS patients. Similarly, a case report by Asano et al. demonstrates that the combination of neutralizing antibodies (nivolumab for PD-1 and ipilimumab for CTLA-4) attenuates bone metastasis in a renal cell carcinoma (RCC) patient. A fracture in the humerus diaphysis with osteolytic changes due to bone metastasis and lung metastasis. No sign of tumor or metastasis in the histopathological examination after four courses of ICIs treatment.

As a primary goal of this Research Topic to improve knowledge and significant advancement of bone metastasis in osteoimmunology, Kähkönen et al. summarize and short-listed 24 anti-bone metastatic therapies by utilizing a novel 1stOncology database based on osteoimmuno-oncology (OIO) concept. This OIO approach deals with the interaction patterns between cancer, bone, and immune cells. Twenty drugs were short-listed from 1498 for breast cancer and 746 for prostate cancer. The authors provide an innovative approach and robust platform to identify therapies for immune activation and prevention or attenuation of bone metastasis in breast and prostate cancer.

Simultaneously, the collection of articles in this Research Topic splendidly adds novel procedures, attractive targets, and ideas to combat bone metastasis in the milieu of Osteoimmunology. Indeed, this Research Topic will improve our knowledge of bone metastasis and related events in various cancers.

## Author contributions

JS: Funding acquisition, Supervision, Writing – original draft, Writing – review & editing. GS: Writing – original draft, Writing – review & editing. MP: Writing – review & editing.
